# Advanced gastrointestinal stromal tumor patients benefit from palliative surgery after tyrosine kinase inhibitors therapy

**DOI:** 10.1097/MD.0000000000009097

**Published:** 2018-01-12

**Authors:** Hai-Bo Qiu, Zhong-Guo Zhou, Xing-Yu Feng, Xue-Chao Liu, Jing Guo, Ming-Zhe Ma, Ying-Bo Chen, Xiao-Wei Sun, Zhi-Wei Zhou

**Affiliations:** a Department of Gastric and Pancreatic Surgery; b State Key Laboratory of Southern China, Department of Hepatobilliary Oncology, Sun Yat-Sen University Cancer Center, Collaborative Innovation Center for Cancer Medicine; c Department of General Surgery, Guangdong General Hospital, Guangzhou, Guangdong, China.

**Keywords:** advanced gastrointestinal stromal tumor, outcome, surgery, TKIS therapy

## Abstract

The role of palliative surgery is controversial in advanced gastrointestinal stromal tumors (GIST) after tyrosine kinase inhibitors (TKIs) therapy.

We evaluated safety and clinical outcomes in a single institution series of advanced GIST patients from January 2002 to December 2008.

One hundred and fifty-six patients had been recruited, including 87 patients underwent surgical resection and 69 patients kept on TKIs treatment. Four patients had major surgical complications. Median follow-up was 38.3 months, the overall survival (OS) and progression-free survival (PFS) of the patients in surgical group were longer than the nonsurgical group, PFS: 46.1 versus 33.8 months (*P* < .01), OS: 54.8 versus 40.4 months. In the subgroup analysis for the patients received surgery, the median PFS for patients with progression disease, stable disease, and partial response was 33.3, 51.5, and 83.0 months, respectively (*P* < .01). Median OS was 68.0 months in those with only liver or peritoneal metastases, and 45.3 months in those with both metastases. Median PFS of patients underwent R0/R1 resection was 73.6 months compared with 35.8 months in R2 resection patients (*P* < .01).

Patients with advanced GISTs have prolonged OS after debulking procedures. Surgery for patients who have responsive disease after TKIs treatment should be considered.

## 1 Introduction

Gastrointestinal stromal tumors (GISTs) are the most common sarcoma of the gastrointestinal (GI) tract, GISTs can arise anywhere along the GI tract, the most common primary sites are stomach (60%) and small intestine (30%).^[1]^ Surgery is the primary treatment for patients with localized GIST, which can be surgically resected, but over 40% of cases may recur and metastasis.^[2]^ Historically, the outcome of patients with unresectable primary or metastatic GISTs were poor, with a median survival of 1.5 years approximately.^[3]^ Development of targeted tyrosine kinase inhibitor (TKIs) therapies that have revolutionized the treatment and dramatically improved the clinical outcomes after elucidation of GIST molecular biology as a mutation-driven cancer.^[4,5]^ About 85% of GISTs are caused by gain-of-function mutations in KIT or Platelet-derived growth factor receptor alpha (PDGFRA).^[6,7]^ Imatinib mesylate, a relatively selective TKI of KIT, PDGFRA, and BCR-Abl, improves prognosis of patients with GIST as therapy for advanced disease significantly.^[8]^ However, approximately 15% of patients were primary resistant to imatinib therapy,^[4,9]^ and over 80% of patients eventually develop progression disease (PD) because of secondary-resistance mutations in additional KIT exons.^[10,11]^ Afterwards, another drug has been shown definitive clinical benefit in GIST following resistance to imatinib was sunitinib malate.^[12]^However, resistance to sunitinib subsequently evolve, generally within 1 year of treatment. The third drug for GIST, Regorafenib has been confirmed that significantly improved progression-free survival (PFS) and disease control rate in patients with advanced GIST progressing after failure of at least imatinib and sunitinib.^[13]^

Many centers have considered adding surgery to TKIs therapy for patients with metastatic GIST. The rationale is reduction of tumor burden might decrease the risk of secondary resistance or operation for focal progression lesions which may have already developed secondary resistance. Here, we report the clinical outcome of 156 patients with metastatic GIST, the largest series so far, who were treated with TKIs and then underwent surgery.

## 2 Methods

### 2.1 Patients and preoperative treatment

This retrospective study was approved by the institutional review boards of Sun Yat-Sen University Cancer Center, Guangzhou, China. All methods and manipulations were performed in accordance with the requirements of this license. The study population consisted of 156 consecutive patients (106 men and 50 women) from January 1, 2002 to December 31, 2008 at Sun Yat-Sen University Cancer Center. Pathologic material was examined and the diagnosis was confirmed using standard hematoxylin/eosin staining and CD117 immunohistochemistry on formalin-fixed, paraffin-embedded tissue as previously described.^[14]^

These patients were referred to the sarcoma service at the Sun Yat-Sen University Cancer Center, where their care was managed by a multidisciplinary team, including medical oncologists, surgical oncologists, pathologists, imaging specialists. All patients were surgical candidates with good performance status and response to treatment, medical recommendations to proceed with surgery were made jointly by the multidisciplinary team and given to the patients who decided whether to surgery or not.

Clinical response was defined upon the absence of disease according to either Response Evaluation Criteria in Solid Tumors or Choi criteria in computed tomography imaging or magnetic resonance imaging.^[15,16]^ Patient data were collected and recorded in a database.

Patients were retrospectively divided in 2 groups. Surgery group (n = 87) comprises patients who were operated after molecular therapy, nonsurgery group (n = 69) comprises patients kept on TKIs therapy without carrying out surgical resection.

### 2.2 Surgery

All surgical procedures were performed at the Sun Yat-Sen University Cancer Center. All patients discontinued use of systemic therapy 1 to 14 days before elective surgery. The general approach of surgery was trying to remove all sites of disease in patients with responsive disease and all sites of progressing disease in the other patients, while reserving function to the greatest extent possible. Most patients underwent further removal of as much additional disease tissue as possible once progressing disease was removed, given the constraints provided by the patient's overall health and the location and extent of disease. Postoperative systemic treatments were given by the multidisciplinary team.

### 2.3 Pathologic and molecular analysis

Postimatinib surgical specimens were carried out for assessment of pathologic response and were based on microscopic findings. For molecular analyses, the most representative areas of primary and metastatic tumors were selected. Mutational analyses were performed on genomic DNA extracted from paraffin embedded or fresh frozen tumor tissue using a combination of polymerase chain reaction amplification, denaturing high-performance liquid chromatography screening, and automated sequencing, as described previously.^[17]^ Samples were molecularly characterized by carrying out DNA sequencing of exons 9, 11, 13, and 17 of c-KIT gene and of exons 12 and 18 of PDGFRA.

### 2.4 End points and statistics

PFS was calculated as the length of time from the beginning of first-line imatinib treatment for metastatic disease to the date of documented progression, recurrent disease or death from any cause, whichever occurred first. Overall survival (OS) was defined as the length of time from the time of first imatinib for metastatic disease or the date of surgery for metastatic disease to death from any cause.

Statistical analyses were performed using SPSS 13.0. Differences among variables were assessed by chi-squared analysis or 2-tailed Student's *t* tests. Data are presented as the mean ± standard deviation unless otherwise indicated. OS and PFS curves were calculated by the Kaplan–Meier method, and the differences between the 2 groups were compared by log-rank test. A *P* value <.05 was considered statistically significant.

## 3 Results

We recruited 156 advanced GIST patients between January 2002 and December 2008. Of the 156 patients, 87 (55.8%) patients received palliative resection, whereas 69 (44.2%) patients continued therapy with TKIs. The median age of all patients was 58.3 years (27–85 years) and 34% were women. No statistical significant differences were observed between the 2 groups with regard to sex and age. In the group of patients with surgery, the primary tumors originated mostly in the stomach (63.3%) or small intestine (26.4%). Similarly, 68.2% patients’ primary site was in stomach and 14.5% was in small intestine for the patients without surgery. Besides, there was no difference in genotype between 2 groups, the most common mutation was found in c-KIT exon 11, the percentage was 66.7% and 63.7%, respectively in surgery group and nonsurgery group. Demographic data for the patients in 2 groups are presented in Table 1.

**Table 1:**
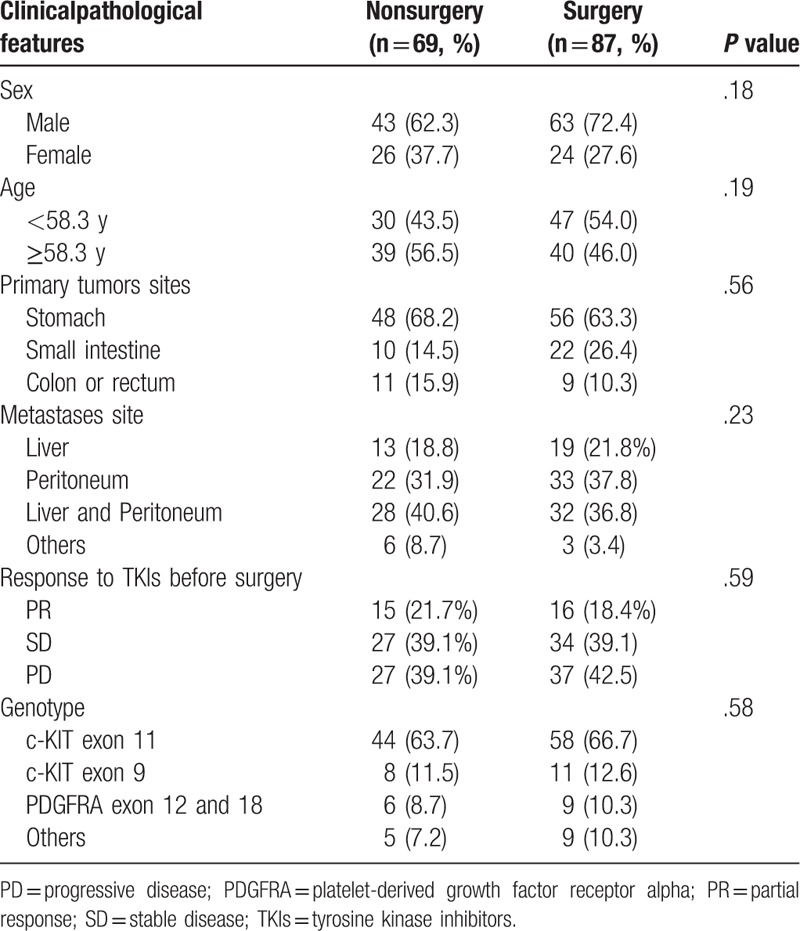
Comparison of clinicopathological features between the patients in the nonsurgery and surgery groups.

We performed surgery in 87 patients with metastatic GIST after treatment with TKIs. The best response during preoperative TKIs therapy was partial response (PR), 16 of 87(10.3%) patients had PR before surgery, 34 patients (21.8%) had stable disease (SD), and 37 patients (23.7%) had disease progression. At the time of surgery, all patients were being treated with imatinib mesylate (400–800 mg/d), except for 7 patients who had been switched to sunitinib. The median time of preoperative molecular therapy was shorter in patients with responsive disease (15 months) than in those with resistance (27 months). All patients kept on taking TKIs therapy after surgery.

### 3.1 Surgical outcomes

Surgical procedures performed are listed in Table 2, the most common procedures were gastrectomy with or without splenectomy, followed by gastrectomy with bowel resections, with or without hepatic metastectomy. Removal of multiple omental or peritoneal tumor nodules by omentectomy or limited peritoneal stripping was performed in 43 patients (62%). The overall 30-day postoperative complication rate was 4.6% (4 of 87 patients). Two patients required reoperation for postoperative bleeding. Two patients were reexplored for early anastomotic leaks after gastrectomy. The median blood loss was 270 mL, median hospital stay was 8 days. There were no perioperative deaths.

**Table 2:**
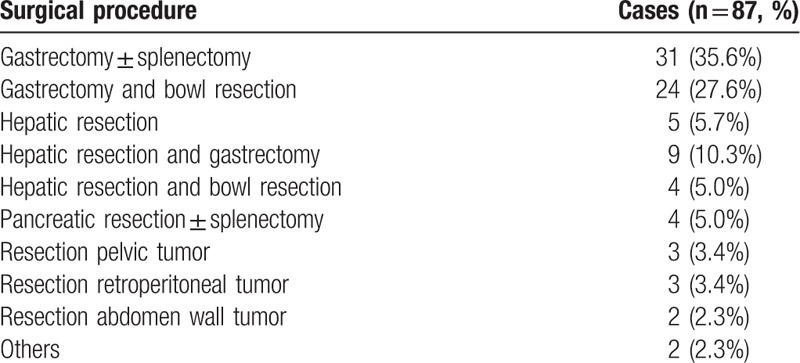
Surgical resection characteristics.

Surgical outcome correlated strongly with the disease status of the patient before surgery (Table 3; *P* < .01). Following surgery, all patients (16 patients, 100%) with PR before surgery underwent R0/R1 resection, compared with 64.7% of patients with SD and 35.1% of patients with PD, respectively. Bulky residual disease remained following surgery (R2 resection) in 0%, 35.3%, and 64.9% of the patients with PR, SD, and disease progression, respectively.

**Table 3:**
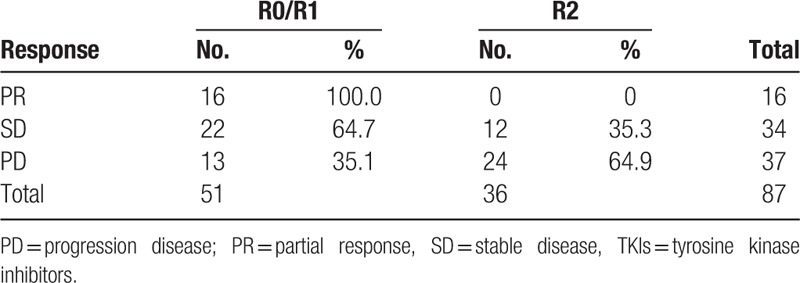
Surgical outcome according to disease response to TKIs therapy before surgery.

### 3.2 Survival outcomes

**Figure 1:**
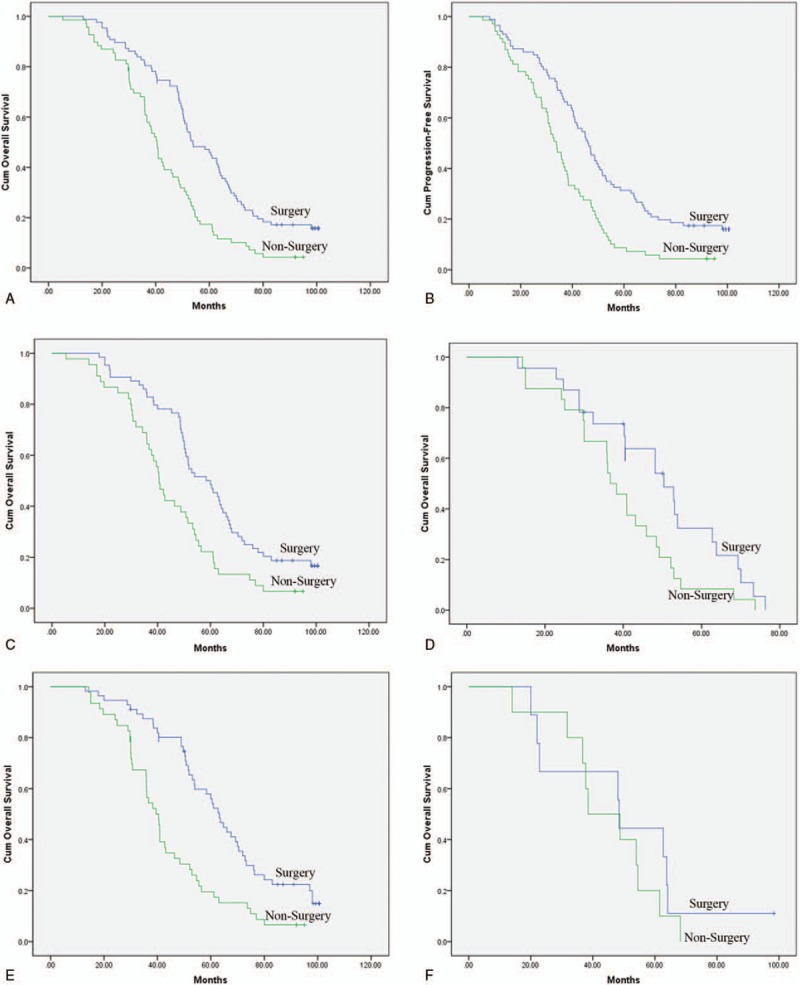
Survival outcome comparison for the patients in surgery group and nonsurgery group. (A). Overall survival of all 156 patients comparison between the patients in surgery group and nonsurgery group. (Median OS: 54.8 mo vs 40.4 mo, *P* < .01). (B). The difference in PFS between patients in these 2 different groups. (Median PFS: 46.1 mo vs 33.8 mo, *P* < .01). (C). Overall survival comparison of patients with primary tumor site in stomach, 2-year OS was 87.0% in surgery group, and 83.3% in nonsurgery group, respectively, (*P* < .01). (D) The difference in OS for the patients with primary tumor site in nonstomach, 2-year OS: 89.1% versus 82.2%, *P* < .01. (E) Patients with KIT exon 11 mutated GIST resection had longer (63.2 mo) median overall survival compared with patients without palliative surgery (39.5 mo), *P* < .01. (F) There was no difference in OS for the patients with exon 9 mutation, (*P* > .05). GIST = gastrointestinal stromal tumors; OS = overall survival; PFS = progression-free survival.

The median follow-up time was 23.7 months (3–81.5 months). Both OS and PFS for patients in the surgery group were significantly longer than those in the nonsurgery group. The median PFS of patients were 46.1 months in surgery group and 33.8 months in nonsurgery group, 2-year PFS rate were 89.7% and 85.5%, respectively, *P* < .01 (Fig. 1A). Similarly, there was a significant difference in median OS between the surgery group and nonsurgery group: 54.8 versus 40.4 months, *P* < .01 (Fig. 1B).

In the subgroup analysis, first according to the primary tumor site, all 156 patients were divided into 2 groups: the patients with primary tumor in stomach (109 patients, 66.7%) or nonstomach (47 patients, 33%). There was a significant difference in OS between surgery group and nonsurgery group no matter where primary tumor site is. Patients with primary tumor site in stomach, OS was longer in the patients with surgery (61 months) compared with the patients without surgery (41.6 months). Two-year OS rate were 87.0% versus 83.3%, *P* < .01 (Fig. 1C). Patients with nonstomach primary site had a median OS of 50.0 months in surgery group and 35.6 months in nonsurgery group, with a 2-year OS of 89.1% and 82.2%, respectively, (*P* < .01, Fig. 1D).

Then the patients’ survival outcome was analyzed by different genotype. Patients with KIT exon 11 mutated GIST resection of metastatic disease was also associated with significantly longer (63.2 months) median OS compared with patients without palliative surgery (39.5 months). Two-year OS of 92.9% and 87.0%, respectively, (*P* < .01, Fig. 1E). However, there was no difference in OS between surgery group and nonsurgery group for the patients with KIT exon 9 mutation, OS were 65.9 and 54.4 months, respectively, (*P* > .05, Fig. 1F).

**Figure 2:**
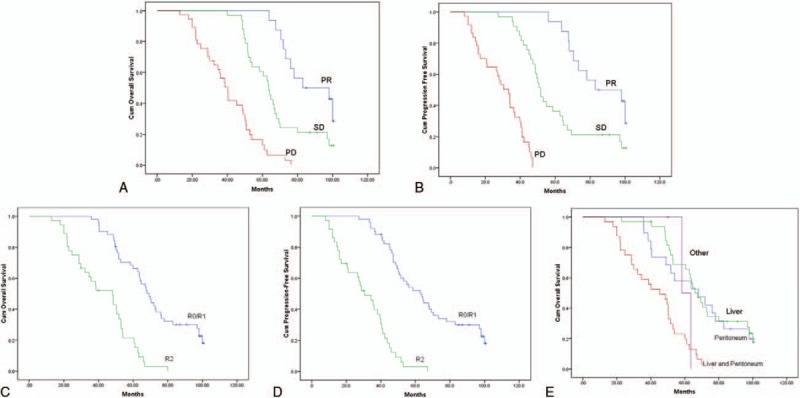
(A) Patients with responsive TKIs treatment before palliative surgery experienced significantly better OS than those with progression disease (*P* < .01) (B). The median PFS for patients with progression disease, stable disease, and partial response was 33.3 months, 51.5 months, and 83.0 months, respectively (*P* < .01). (C). Median OS of patients underwent R0/R1 resection was 62.7 months compared with 29.4 months of the patients with R2 resection (*P* < .01). (D) Median PFS was 73.6 months versus 35.8 months (*P* < .01) in patients. OS = overall survival; PFS = progression-free survival; TKIs = tyrosine kinase inhibitors.

Patients with responsive TKIs treatment before palliative surgery experienced significantly better OS than those with PD (*P* < .01, Fig. 2A). Fourteen patients have died of disease, and no patient has died of another cause. Patients with PR and SD both had 2-year survival of 100%, which was significantly longer than that of the group with PD. Patients with PD had a median survival of 40.0 months, and a 2-year survival of 75.7%, respectively. All of the patients presenting with PD demonstrated poorer survival compared with the patients who had responsive TKIs treatment before palliative surgery. The median PFS for patients with PD, SD, and PR was 33.3 months, 51.5 months, and 83.0 months, respectively (*P* < .01, Fig. 2B).

Furthermore, OS data of patients was compared in whom macroscopically complete resection could be achieved (R0/R1) and those with residual tumor (R2). Median OS was 77.5 months for the R0/R1 group compared with 48.3 months in the R2 group (*P* < .01; Fig. 2C). The median PFS was 73.6 months versus 35.8 months (*P* < .01) in patients underwent R0/R1 and R2 resection, respectively (Fig. 2D).

The outcome of patients depending on metastatic organ involvement was then compared. In the overall population, median OS was 68.0 months who were affected only by liver metastases, 67.6 months in those with peritoneal disease, and 45.3 months in those with both liver and peritoneal metastases (Fig. 2E; *P* < .01).

## 4 Discussion

Complete surgical resection followed by adjuvant imatinib therapy is the standard of treatment strategy for localized GIST in intermediate and high-risk patients. Currently, the management of advanced GIST is to escalate the dosage of imatinib or switch to second-line sunitinib therapy for patients who progressed on imatinib.^[12,18,19]^ However, the role of surgery for advanced GISTs is not well established yet. No definitive data exist to prove whether surgical resection improves clinical outcome in addition to TKIs therapy for patients with metastatic GIST.

Over 80% of patients treated with TKIs induced PR or SD, which has become the primary treatment of metastatic GISTs.^[8,20]^ However, there are several reasons to consider surgical resection in patients with metastatic GIST who are being treated with target therapy. Although TKIs control tumor growth in the majority of patients, complete responses are rarely achieved. In the present study only 3 patients had no viable cancer cells upon microscopic examination of the surgical specimen. Besides, even tumors had good response to molecular therapy appear nonviable by radiologic imaging usually still harbor alive cells, which often have KIT protein activation.^[21]^ Furthermore, it has been proved that most cases who initially respond to TKIs develop resistance eventually. The median time to progression is approximately 2 years.^[20]^ The most common mechanism of resistance to TKIs is secondary mutations in driver genes.^[9,22]^ Once resistance develops, there is a small chance of controlling patient's disease. Imatinib dose escalation in 133 patients who progressed on 400 mg/d resulted in a median PFS of only 81 days, with 18% progression free at 1 year.^[18]^ Meanwhile, patients switched to sunitinib will have progression free for approximately 26 weeks.^[12]^

Based on the above rationale, a combination of molecular therapy and surgery for the treatment might benefit for patients with advanced GIST. Previously, a few small series retrospective studies have demonstrated survival benefit in advanced GIST patients responding to imatinib followed by underwent cytoreductive surgery.^[23–26]^ However, there was no publication yet compared all the metastatic GIST population between surgery or nonsurgery directly.

This analysis focuses on the outcome of a large series of patients who underwent surgical resection of advanced GIST following TKIs, including imatinib and sunitinib therapy. The aim of surgical resection was to remove either single lesions that had already developed resistance to stop disease progression or the whole tumor bulk to prevent subsequent resistance to TKIs.

Our results showed both OS and PFS for patients in the surgery group were significantly longer than those in the nonsurgery group. The median PFS of patients were 46.1 months in surgery group and 33.8 months in nonsurgery group, 2-year disease-free survival rate were 89.7% and 85.5%, respectively. Similarly, there was a clear difference in median OS between the surgery group and nonsurgery group as well: 54.8 versus 40.4 months. This is the first study directly comparing all advanced GIST patients’ survival based on simple classification of the patients into surgery and nonsurgery groups, which might indicate that the long survival achieved in patients received the treatment with a combination of molecular therapy and surgery.

Genome type analysis was finished in the follow-up as it was not routine at the time of surgery for all patients. In the subgroup analysis, we found that patients with c-KIT exon 11 rather than exon 9 as primary mutation benefit from the cytoreductive surgery. These data are consistent with a previous report from Europe.^[27]^ However, because the patient population (19 patients) of this series is not large enough, is it worthwhile to carry out palliative surgery in the advanced GIST patient with c-KIT exon 9 mutation is still unknown.

Surgical debulking combined with drug therapy resulted in a median OS of 61.0 months, comparing with 41.6 months in nonsurgery 2-year OS rate were 87.0% versus 83.3% in patients with primary tumor site in stomach. Similar results were seen in non-stomach primary tumor site patients. Thus, our data suggest that patients may benefit from palliative surgery, no matter where the primary tumor site is.

Patients who have responsive disease to TKIs therapy may benefit from elective surgical resection, our results showed that patients with PR and SD both had 2-year survival of 100%, which was significantly longer than the PD patients. The median PFS for patients with PD, SD and PR was 33.3 months, 51.5 months, and 83.0 months, which means patients with responsive disease may benefit from debulking procedures, particularly if surgery achieves either complete extirpation of all tumors or reduction in tumor burden increase the R0/R1 resection rate (Table 3), this is in concordance with previous analyses.^[23,24]^ However, our patients have a higher response rate (PR + SD >50%) to TKIs, and a larger part of them were all judged resectable, whereas in the other series, only 7% of patients were considered suitable for surgery. Such direct comparisons between studies have to be interpreted with much caution as selection bias originate from potential differences in the study populations.

In our analysis, patients with R0/R1 surgery had a median OS of 77.5 compared with 48.3 months in those with incomplete R2 resection (Fig. 2C), a survival benefit of more than 2 years. Complete resection had a significant positive prognostic value in the multivariate analysis.

Of note, patients with disease limited to the liver showed the longest median OS in this study. We believe that this data does not allow an unequivocal recommendation for surgery but that it does support a surgical approach in metastatic lesions in carefully selected patients, especially with metastases restricted to 1 organ.

We here present by far the largest series of patients study with palliative surgery in advanced GIST, our data provide evidence that resection of metastatic disease after TKIs therapy is associated with a long median OS. Quality of life after surgery was not investigated, however, we did not observe surgery-related mortality are worse than previous trials with TKIs as a single therapy.

## 5 Conclusion

Advanced GIST patients with good performance status and response to treatment benefited from palliative surgery. Prospective phase III studies are ongoing to assess whether or not surgical resection improves outcome in patients with advanced GIST responding to TKIs therapy.
